# How to Evaluate Non-Growing Cells—Current Strategies for Determining Antimicrobial Resistance of VBNC Bacteria

**DOI:** 10.3390/antibiotics10020115

**Published:** 2021-01-26

**Authors:** Susanne Fleischmann, Christian Robben, Thomas Alter, Peter Rossmanith, Patrick Mester

**Affiliations:** 1 Institute of Food Safety and Food Hygiene, Department of Veterinary Medicine, Freie Universitaet Berlin, 14163 Berlin, Germany; Susanne.Fleischmann@fu-berlin.de (S.F.); thomas.alter@fu-berlin.de (T.A.); 2 Christian-Doppler Laboratory for Monitoring of Microbial Contaminants, University of Veterinary Medicine Vienna, 1210 Wien, Austria; christianrobben@ymail.com; 3 Joint BioEnergy Institute, Lawrence Berkeley National Laboratory, Berkeley, CA 94720, USA; peter.rossmanith@vetmeduni.ac.at; 4 Unit of Food Microbiology, Institute of Food Safety, Food Technology and Veterinary Public Health, University of Veterinary Medicine Vienna, 1210 Wien, Austria

**Keywords:** viable but non-culturable, VBNC, antimicrobial resistance, antibiotic resistance, tolerance, bacterial viability, live/dead, viability PCR

## Abstract

Thanks to the achievements in sanitation, hygiene practices, and antibiotics, we have considerably improved in our ongoing battle against pathogenic bacteria. However, with our increasing knowledge about the complex bacterial lifestyles and cycles and their plethora of defense mechanisms, it is clear that the fight is far from over. One of these resistance mechanisms that has received increasing attention is the ability to enter a dormancy state termed viable but non-culturable (VBNC). Bacteria that enter the VBNC state, either through unfavorable environmental conditions or through potentially lethal stress, lose their ability to grow on standard enrichment media, but show a drastically increased tolerance against antimicrobials including antibiotics. The inability to utilize traditional culture-based methods represents a considerable experimental hurdle to investigate their increased antimicrobial resistance and impedes the development and evaluation of effective treatments or interventions against bacteria in the VBNC state. Although experimental approaches were developed to detect and quantify VBNCs, only a few have been utilized for antimicrobial resistance screening and this review aims to provide an overview of possible methodological approaches.

## 1. Introduction

**Figure 1:**
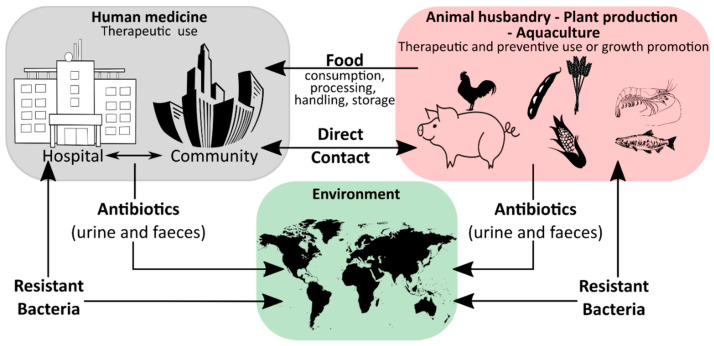
A schematic overview of the ecology of antibiotics, showing how these drugs are circulated between different environments, such as the medical environment, agricultural settings, the aquacultural environment, the pharmaceutical industry and the wider environment, adapted from [9].

Humanity′s history is a continuous battle between us and microbial pathogens and for the most part, we were on the losing side with bacterial and viral infections being among the major causes of morbidity and mortality worldwide. Thanks to the development and improvement of sanitation, hygiene practices, and especially the discovery of antibiotics and vaccinations since the early 20th century, deaths from infectious diseases have declined markedly which can be considered one of the biggest success stories in human history [1]. However, although the burden of disease has been drastically reduced, public health threats posed by microbes are still present and new challenges demand new solutions. Over the last years, an increase in infectious diseases has been observed in almost all countries, regardless of their economic development, with a rising incidence of foodborne illness and nosocomial infections [2,3,4,5]. For effective prevention and intervention measures, the assessment of microbial viability is crucial in determining the safety of food and drinking water, as well as the environmental and medical sector. Together with the rising drug resistance of pathogens against traditional antibiotic therapies, the WHO has defined this problem as one of the most serious public health challenges [6,7,8]. Figure 1 highlights the use of antibiotics in human medicine, animal husbandry, agriculture, and the subsequent circulation between these applications and the environment leading to an increase in the prevalence of antibiotic resistant bacteria in different ecological niches.

During the last decades, a plethora of antibiotic resistant bacterial genotypes have been reported and as a result, research has intensified to identify novel antibacterial agents and antibiotics [9,10,11]. While coming to the fore recently, the impact of phenotypic plasticity on antimicrobial and antibiotic resistance has been far less studied. Phenotypic plasticity describes the ability of bacteria to change their phenotype (in this case their antimicrobial resistance profile) without underlying genetic changes. While phenotypic plasticity in bacteria has been known for a long time already, the understanding of the underlying adaptive and regulatory processes, as well as their role in the infectious process has only recently been significantly expanded [6,12].

In order to preserve viability and adaptability in constantly changing environmental conditions, bacteria have developed various mechanisms for switching from a vegetative to a metabolically inactive, but highly resistant and persistent state. The most studied type of such cellular dormancy is sporulation of sporogenous bacteria, while in recent years, the viable but non-culturable (VBNC) state for many bacterial pathogens has gained more attention [12,13,14,15,16]. The VBNC state is a condition of reduced metabolic activity, induced in response to conditions of stress. Entry into the VBNC state can be facilitated by a number of environmental factors, such as a lack of nutrients, temperature shifts, or the presence of antibiotics, disinfectants, and sanitizers [12,17,18,19]. When a microorganism enters the VBNC state, both the morphology and physiology of the cell changes to allow its survival under adverse conditions. Then, once the environmental conditions return to normality, the cell can get back to its culturable state, through a reactivation process, which is referred to as “resuscitation”.

**Figure 2:**
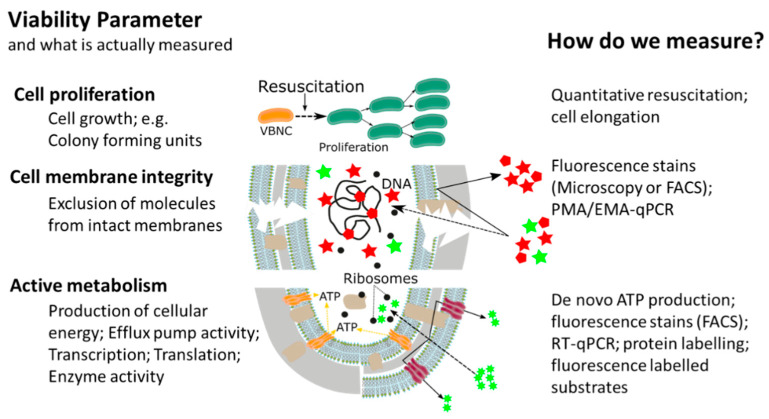
Overview of currently available methods for measuring different bacterial cell viability parameters, adapted from [34].

This widespread, or almost universal, occurrence of bacterial cellular dormancy can lead to serious diagnostic and safety problems. Bacteria in the VBNC state represent a diagnostic challenge, due to the simple fact that the majority of current tests are either entirely culture based or need at least one cultivation step, thus failing to detect non-growing VBNC cells [15,20,21,22]. As a result, the absence of growth in culture-based methods does not always mean non-viability, but can also be interpreted as: (i) Incorrect culture medium; (ii) stress or damage of cells leading to a dormant state, (iii) low population density thus leading to no visible growth; and (iv) slow-growing cells and no visible growth. We want to emphasize that the reason for our ongoing dependency on culture-based methods is of course their proven efficiency and cost-effectiveness as diagnostic tools for many years. However, given their inability to detect dormant cells, it is important to point out that the growth and division of cells is not the only accepted parameter in assessing bacterial viability. In addition to reproduction, metabolic activity as well as an intact cell membrane are accepted general parameters for viability assessment of microorganisms, with the latter two having the advantage of being growth independent (Figure 2) [12,23,24]. While the issues of non-culturability, natural occurrence, induction, and resuscitation of the VBNC cell, as well as the underlying molecular mechanisms and the detection of dormant cells from environmental samples are of extreme importance, these topics have been recently reviewed [15,17,24,25] and therefore, will not be the main focus of this article. Rather, in this article, we want to elucidate one of the major problems associated with dormant cells, namely their high tolerance and resistance against a wide range of antimicrobials, biocides, and antibiotics [12]. Their elevated tolerance enables cells in the VBNC state to withstand antimicrobial interventions in medical, as well as food-processing environments allowing resuscitation from the VBNC state and possible recontamination of those environments [26,27,28,29,30,31,32]. Phenotypic plasticity needs to be considered when performing antimicrobial efficacy testing. It is clear that the evaluation of antimicrobial treatments or interventions solely on cells in the logarithmic growth phase, combined with subsequent growth based quantification methods, has some significant blind spots which need to be addressed to ensure public health in the medical, as well as food production sector [20,21,33]

Although antimicrobial resistance is generally recognized as one of the major challenges posed by VBNC cells, the overall number of studies investigating this effect remains sparse. It is our opinion that the main reason for this is the technical and experimental difficulty of investigating antimicrobial resistance of dormant cells in a quantitative way. Therefore, this article aims to provide an overview of the currently available approaches, which could be used for antimicrobial effectivity testing of bacteria in the VBNC state.

## 2. Current Explanations of Antimicrobial Resistance of Bacteria in the VBNC State

In addition to the special case of sporulating bacteria, there are a few different manifestations of phenotypic plasticity in microbes resistant to traditional antibiotic therapy: L-forms (L-transformation, cell wall deficient bacteria, CWD), persister cells, and VBNC bacteria [6]. While it is often hard to draw a clear line between the two states of cellular dormancy, VBNC and cellular persistence, the latter is a more broader term and has been recently reviewed extensively [14,18]. Although many of the methods and experimental approaches described in this article could be also utilized to investigate the antimicrobial resistance of persister cells, as previously mentioned, this article focuses on bacteria in the VBNC state.

When talking about dormant or VBNC cells, a lot depends on the perspective with which we approach this topic. VBNC cells are defined as microbes that fail to cultivate on routine media on which they usually grow and yet are still alive, a phenomenon that has been first described by Xu et al. in 1982 for *Vibrio* (*V.*) *cholerae* [12,35,36]. Thus, the VBNC state could either be described as a “failure of the culture dependent technique” (as in hindering the bacterial growth), or as a dormant state in which the proliferation of the microbes is blocked until they get a “wake up call” [19,37]. As is so often the case when using such broad terminology as dead, alive, or dormant, it is not easy to draw simple singular conclusions.

For instance, for *V. vulnificus* the VBNC state can be categorized as a failure of the growth-based approach. In this case, researchers found that as a response to storage at 4 °C without nutrients, the bacteria significantly reduce their metabolism and important enzyme pathways are effectively downregulated [38,39]. One of these downregulated pathways is connected to the resistance against oxidative stress such as H_2_O_2_. Consequently, *V. vulnificus* cells lose the ability to withstand naturally occurring H_2_O_2_ concentrations present in standard growth media, resulting in their non-culturability. Recognizing this effect has immediately led to the addition of either catalase or pyruvate to the growth medium, reducing the effective H_2_O_2_ concentration, and thus allowing the culturability of “VBNC” cells [39].

However, such “failures of the culture-dependent approach”, as described above, are by far the exception regarding VBNC induction and resuscitation. More often, exposure to either unfavorable environmental conditions or significant stress induces a dormant state that cannot be reversed through media adaptations or, as is done in typical antimicrobial efficacy testing, by removing the respective stress condition or addition of a neutralizer. Unfortunately, little is known about the underlying molecular mechanisms, but one well described system for the VBNC/dormant form of bacteria was triggered by the discovery of type II toxin-antitoxin systems (TAS), first described for the *hipA* gene in *Escherichia* (*E.*) *coli* [40,41]. TAS are thought to be a general mechanism of genetic control over the formation of dormant bacteria and also play an important role in the formation and organization of biofilms, in which the VBNC state also often occurs [14]. These systems usually contain two genes encoding for the stable toxin and the unstable antitoxin, the latter being sensitive to degradation by cellular proteases [42]. Under normal conditions, the toxin and antitoxin are bound to each other, forming the non-toxic complex. Under stress, caused by adverse environmental conditions, antitoxins are degraded. The released toxin causes a sharp decrease in translation, replication, and cell growth inducing persistence, as well as the VBNC state thus dramatically increasing the antimicrobial resistance of the bacteria [42,43]. It has been reported that TAS are widely spread among bacterial chromosomes and recently a detailed review regarding TAS has been published by Andryukov et al. [6]. In addition to TAS systems, there are also other regulators known, such as *rpoS* or *oxyR*, that control the bacterial VBNC state as a response to stress exposure [12,44]. RpoS is a sigma factor known to control up to 10% of the *E. coli* genome and plays an important role in the survival of bacteria in the stationary phase, as well as under adverse conditions. *OxyR* and some other regulated genes, are one of the most important regulation systems in the oxidative stress response. However, it has to be pointed out that their role in VBNC induction and resuscitation is still largely unknown [12]. What these regulators and TAS have in common, is the fact that they are an immediate response to adverse environmental conditions. Therefore, this response can be seen more as a part of the phenotypic plasticity of bacteria in comparison to genetic antimicrobial resistance mechanisms such as efflux pumps or membrane modifications [6,43,45,46]. Indeed, it seems to be the combination of phenotypic changes with a low metabolic activity of cells in the VBNC state, which causes their elevated resistance, while still being able to resuscitate and initiate infection. Given their potential health hazard, it is paramount that we learn more about the underlying molecular mechanisms and regulatory pathways [12,26,47,48].

## 3. Methodological Approaches for Antimicrobial Effectivity Testing of VBNC Bacteria

Given their impact and potential health hazard, a correct evaluation of antimicrobial effectivity against VBNC bacteria is crucial, but necessitates improving our methodological approach, which is predominantly growth based. This article aims to provide an overview of the currently available methodological approaches, which can be used for antimicrobial effectivity testing for bacteria in the VBNC state. While there is a certain overlap regarding methods used for the detection and antimicrobial effectivity testing of VBNCs, we will not include methods that can currently be used only for the detection of VBNC bacteria as these have been recently reviewed [24]. As previously outlined, cell growth, metabolic activity, and cell membrane integrity are accepted parameters for assessing the viability of microbes with the latter two having the advantage of being growth independent (Figure 2) [12,23,24]. Therefore, it comes as no surprise that current methods for studying VBNCs aim at detecting one of the three parameters (Figure 2). In terms of antimicrobial effectivity testing capability, different methodological approaches for assessing the respective viability parameters have been published and their background, advantages, and limitations will be detailed in the respective sections.

In this review, we focus on the applicability of the presented methods for antimicrobial effectivity testing of VBNC cells with standard lab equipment and therefore, very laborious and time-consuming, as well as methods which require complex, rare, and expensive equipment were excluded. This includes mRNA transcription assays [38,49,50], isothermal microcalorimetry (IMC) [51,52,53,54], Fourier transform infrared spectroscopy [55], or assays which can hardly distinguish between viable and dead cells such as many enzymatic assays.

### 3.1. Cell Proliferation

A successful cultivation is probably the clearest and most accepted indication that an organism is alive while, as has been previously pointed out, an unsuccessful cultivation does not prove the lack or absence of life [23]. Still, microbial culturability is the gold standard to prove viability and therefore, resuscitation of bacterial cells from the VBNC state is the most straightforward experimental approach to perform antimicrobial effectivity testing against VBNCs.

#### Resuscitation

**Figure 3:**
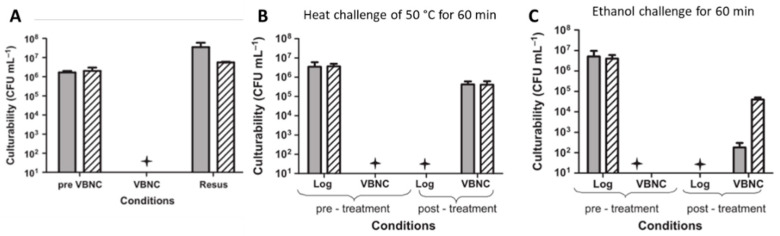
Antimicrobial effectivity screening of *V. vulnificus* (bars represent results of two different strains) in the viable but non-culturable (VBNC) state (adapted from [28]). (**A**) Resuscitation from the VBNC state. Shown is the culturability of the various cell populations examined. “Pre-VBNC” represents log-phase cells, “VBNC” represents culturability of these cells following induction into the VBNC state, and “resus” represents culturability of VBNC cells following resuscitation at 21 °C in artificial seawater. (**B**,**C**) Susceptibility of *V. vulnificus* exposed to different stresses. “Pre-treatment” represents control logarithmic and VBNC cells of *V. vulnificus* not exposed to any treatment. “Post-treatment” represents logarithmic and VBNC cells of *V. vulnificus* subjected to challenge. The star represents plate counts below the detection limit (<10 CFU/mL).

Of special relevance are experimental approaches, in which a complete and reproducible induction into and resuscitation from the VBNC state is possible without a significant CFU/mL reduction (Figure 3A) [28]. A major advantage of the approach is its exclusion of possible regrowth of surviving culturable cells during the resuscitation conditions. Overnight cultures of *V. vulnificus* are diluted 1:100 into ½ strength artificial seawater and then either are immediately exposed to physical or chemical challenges or placed at 5 °C to induce the VBNC state. For resuscitation, *V. vulnificus* cells were simply incubated overnight at room temperature in nutrient-free artificial seawater without the addition of any supplements or growth media. Therefore, the possible regrowth of a number of surviving culturable cells below the detection limit can be excluded [28,35]. Such approach allows antimicrobial effectivity testing against VBNC cells by means of a quantitative CFU/mL reduction after resuscitation, in a similar manner to “normal” efficacy testing with culturable cells. Due to its easy, quick, and quantitative resuscitation, *V. vulnificus* is the perfect model organism for this kind of experiment. After successful induction, VBNC cells can be exposed to varying stress conditions and the CFU/mL log reduction is determined after resuscitation in comparison to the non-treatment control by the standard plate count (Figure 3B,C) [12,28].

In addition to the quantitative resuscitation approaches described for *Vibrio* species, there have been other studies, in which resuscitation has been utilized as a proof of survival of antibiotic treatments. Rivers et al. [56] showed that VBNC *E. coli* were present in mice during urinary tract infections and could be resuscitated after they stopped the antibiotic treatment. The authors demonstrated that culturable pathogenic *E. coli* in mice that were inoculated and received the antibiotic treatment dropped to undetectable levels within 1 week, although a viable population of *E. coli* remained which could be recovered to culturability within 65 days [56]. Pasquaroli et al. [57] managed to resuscitate *Staphylococcus* (*S.*) *aureus* after the vancomycin treatment of biofilms. Different *S. aureus* biofilms were treated with different vancomycin concentrations until a complete loss of culturability, while viable cell populations remained constant. After removal of antibiotic stress, bacteria could be resuscitated in a rich medium supplemented with sodium pyruvate or with a late-log-culture filtrate.

Applicability for antimicrobial effectivity testing:

The methodological approach based on cell proliferation is able to quantify either viable cells or VBNC cells after a successful resuscitation. A successful resuscitation of VBNC cells is defined by the reoccurrence of culturable cells from a VBNC sample in which no culturable cells could be detected before. The approach is not able to detect viable and VBNC cells at the same time from the same sample and dead cells are not actively detected, but rather inferred by a viable cell reduction. Antimicrobial effectivity testing based on cell proliferation of VBNC cells such as resuscitation inhibition or CFU reduction offers the advantage that identical methodological approaches can be used for both culturable and VBNC cells and no additional normalization of analytical methods has to be performed. In order to obtain reliable results based on cell proliferation it is of the utmost importance to distinguish the real resuscitation of VBNC cells from the regrowth of possible surviving “culturable” cells. Ideally, resuscitation should be performed in nutrient poor environments that would not allow the regrowth of surviving culturable cells to levels that would interfere with the interpretation of resuscitated cells.

Limitations:

The main limitation for this experimental approach for antimicrobial effectivity testing against VBNCs is the fact that from over 100 species known to enter the VBNC state, only a small number of pathogens have been successfully and reliably resuscitated [58]. Therefore, it is not surprising that only a few publications are available using resuscitation to specifically analyze the antimicrobial tolerance of VBNC cells. Although resuscitation-based investigations on VBNC tolerance are the most preferential approach, there is a need of alternative methods for tolerance testing of non-resuscitating VBNC pathogens.

### 3.2. Integrity of the Cell Membrane

The existence of an intact and functional cell membrane is considered to be one of the three necessary aspects of microbial viability [23]. In addition to defining each individual cell, the outer cell membrane provides a physical, chemical, and biological barrier against the environment. A compromised membrane would result not only in the entry of harmful chemicals, but also in the leakage of important proteins, cellular energy, or genetic information, and finally in the death of the respective cell. There are different methods available to measure the membrane integrity such as fluorescence staining or PCR based approaches [23], which are being regularly used to determine the viability of cells in the VBNC state and thus will be discussed in detail below.

#### 3.2.1. Fluorescence Microscopy

Several fluorescence-staining procedures were applied to enumerate the total viable bacterial count including VBNC cells in food and environmental samples or pure cultures. In addition to differential fluorescence-staining assays using acridine orange (AO) [59,60,61,62] or a double staining with tetrazolium salt 5-cyano-2,3-ditolyltetrazolium chloride (CTC) and 4,6-di-amidino-2-phenylindole (DAPI) [63,64,65,66], the LIVE/DEAD ^®^ BacLight™ bacteria viability kit, developed by Molecular Probes, is the most common assay to discriminate viable bacterial cells from membrane compromised dead cells [25,67]. Cell membrane integrity is an important hint to identify viable cells. Nevertheless, a combination of different staining methods to additionally measure the metabolic activity is necessary to identify cells in the VBNC state [68,69,70].

**Figure 4:**
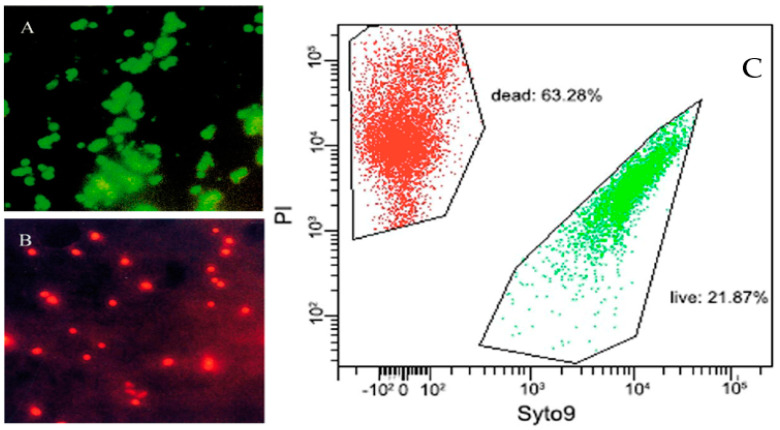
Fluorescence microscopy of viable (**A**) and dead (**B**) *V. cholerae* [68]. VBNC cells adapted from the flow cytometry analysis of VBNC cells (**C**) induced by low temperature storage in beer, adapted from [78]. Live cells (green fluorescence, SYTO 9^®^) and dead cells (red fluorescence, PI) are viewed simultaneously by appropriate excitation and emission spectra.

The BacLight™ is composed of the nucleic acid-binding stains SYTO 9^®^ and propidium iodide (PI) which differ in their spectral characteristics, as well as in their ability to penetrate bacterial cells. SYTO 9^®^ penetrates bacterial membranes resulting in a green fluorescing signal, whereas PI is membrane impermeant and penetrates cells with damaged membranes only. The combination of the two stains results in red fluorescing cells indicating dead cells, while PI reduces the SYTO 9^®^ fluorescence when both stains bind [67,71], enabling the enumeration of viable cells, membrane compromised dead cells, and total counts in a single double staining step (see Figure 4). This kit can be easily used by different detection methods, for example, direct fluorescence microscopy [72,73], microplate reader [68,74,75], flow cytometry [69,76,77,78], or spectroscopic systems such as an optrode [79,80,81]. Robertson et al. [81] published an optimized and faster LIVE/DEAD BacLight™ protocol, in which cultures are grown and stained in the same media without an additional washing step including an adjusted emission spectrum and dye ratio. Another optimized protocol was published by Feng et al. [82] to assess the antibiotic susceptibility for *Borrelia burgdorferi* in comparison with microscopic counting, whereas SYTO 9^®^ was replaced by SYBR Green I.

A single staining method using AO allows also the differentiation of viable green from dead orange cells. AO binds to RNA forming a red-orange fluorescence complex while in connection with DNA a green fluorescence complex is built [62]. For environmental samples such as drinking water, the BacLight™ staining assay produces less background fluorescence as does AO [73]. Therefore, an additional washing step using AO is acquired reducing a rapid detection time [67]. A quantitative analysis of both staining methods can be performed using fluorescence reader systems [27].

The advantages of the BacLight™ staining are that it is a rapid, easy to prepare, and well-studied method to discriminate both viable cells including cells in the VBNC state and membrane compromised dead cells in one-step. The assay can directly and in real time quantify live and dead cells for any given process. To estimate and quantify the bacterial counts, various instruments such as microscopes, microplate reader, and cytometer can be chosen [25,27,67,69,83]. The high degree of contrast between the green color of viable and the red color of dead cells make the results easy to interpret. Studies demonstrate that the BacLight™ staining assay resulted in the highest viable and total bacterial counts compared to other direct count staining methods (AO, CTC and DAPI, or CTC and SYTO 9^®^) [68,73]. Compared to other detecting methods, fluorescent staining in combination with fluorescence microscopy or flow cytometry is easy to use, rapid, sensitive, and visible [83,84]. However, flow cytometry in contrast to fluorescence microscopy, is the more accurate method to estimate bacterial counts down to 2.5% live and 20% dead cells [25,81]. Nevertheless, viable staining methods such as the BacLight™ allows the evaluation of the physiological state of individual bacteria regardless of their species, growth conditions, and treatments including antibiotics [16,19,67]. Using direct fluorescence staining microscopy in combination with the BacLight™ kit, Chaiyanan et al. [68] successfully monitored the membrane integrity of VBNC *V. cholerae* O1 and O139 under chlorine, cooper sulfate, zinc sulfate, and formaldehyde treatment. All treatments showed a greater resistance for VBNC cells in contrast to cells in the exponential phase [68]. Noll et al. [70] induced a VBNC state in *Listeria* (*L.*) *monocytogenes* using benzalkonium chloride and showed a reduced antimicrobial effectivity of benzalkonium chloride, ceftriaxone, gentamycin, linezolid, tetracycline, trimethoprim, and sulfamethoxazole against VBNCs in contrast to their parent cells using flow cytometry. During the study, benzalkonium chloride or the antibiotic substances did not influence the fluorescence properties of SYTO 9^®^ or PI [70].

**Figure 5:**
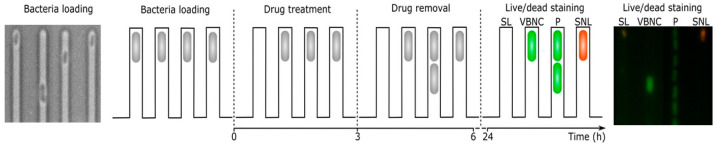
Single-cell microfluidic analysis of susceptible lysed (SL), VBNC (VBNC), persister (P), and susceptible non-lysed (SNL) *E. coli* BW25113 cells before (0 h), during (0–24 h), and after (24 h) the antibiotic treatment combined with SYTO 9^®^ and PI. Live cells (green fluorescence, SYTO 9^®^) and dead cells (red fluorescence, PI) are viewed by time-laps microscopy, adapted from [31].

Bamford et al. [31] monitored *E. coli* cells in the VBNC state via a single-cell approach combined with the BacLight™ staining before, during, and after the ampicillin treatment. Both VBNC and persister cells survived in contrast to susceptible cells that died and lysed during or after the antibiotic treatment (see Figure 5) [31].

Applicability for antimicrobial effectivity testing:

Staining methods to detect the cell membrane integrity in combination with fluorescence microscopy can be applied rapidly in already existing processes with the advantage that no prepended incubation time is necessary. Via viable cell counts, quantitative results can be obtained directly after sampling within half an hour regardless of the bacterial species. Furthermore, the efficiency of bactericides can be direct quantified regarding the cell count difference before and after the treatment of viable cells including cells in the VBNC state using fluorescence microscopy or flow cytometry. Comparing the BacLight™ kit with traditional time intensive culture-based methods, comparable results for viable cells can be achieved, but cells in the VBNC state cannot be detected. Therefore, viable cells which do not grow on agar plates, but are detectable via the BacLight™ kit can be assigned to cells in the VBNC state. Otherwise, the BacLight™ kit only differentiates into viable and dead cells.

Limitations:

The major limitation of the BacLight™ staining is the differentiation between viable and dead cells solely based on bacterial membrane integrity. Therefore, the applicability of the BacLight™ is limited by the decreased membrane permeability or by the insufficient accumulation of the dyes to become detectable [85]. SYTO 9^®^ penetrates all cell membranes in contrast to PI, which only stains nucleic acids in membrane-compromised cells. Therefore, the physiological state of the bacteria affects the binding site of the dyes. For example, chlorine reacts with the cell membrane increasing the membrane permeability and facilitates the penetration of PI. Fluorescing signals from yellow to orange, in addition to the typical signals of red and green are the results and make a clear evaluation more difficult. The intermediate colors are due to the varying amounts of PI, which reflect the different stages of membrane damage [73]. Similar observations were shown by Zhao et al. [86] using fluorescence microscopy after a treatment of VBNC state *E. coli* O157:H7 cells with sonication. Therefore, the total viable cell count was estimated by flow cytometry with regard to the florescence signal of SYTO 9^®^ [86]. With regard to monitoring VBNC cells under the treatment with antibiotics that interfere with the bacterial cell membrane, similar effects as reported for chlorine are possible. Therefore, an underestimation of viable cells may lead to an incorrect conclusion. Contrary to the decreased membrane permeability, there are also cells with an intact cell membrane, but without a metabolic activity [87]. These metabolically inactive dead cells can lead to an overestimation of the viable cell count. Berney et al. [69] described using the BacLight™ staining such an overestimation including intermediate color signals and attributed it to the physiological properties of the cells dependent on the low cytoplasmic membrane permeability next to a metabolic inactive cell. Gram-positive bacteria such as *Enterococcus faecalis* showed no clear differentiation in contrast to Gram-negative bacteria after an UV-A treatment [69]. Cai et al. [88] showed that after an UV-A treatment of *S. epidermis* and *Streptococcus mutans,* as well disagreements using the BacLight™ staining compared to CFU counting and metabolic activity assays. The cell membrane after an UV-A treatment is still intact while the cellular functions are totally lost [88].

In addition, a reaction of chemicals such as chlorine with nucleic acids can also be a reason to alter the binding sites for PI [73]. It is also known, that fluorescence dyes can interact with carbon compounds and preservatives [89].

Given the current literature and possible problems associated with this method, it seems to be advisable that BacLight™ staining based approaches for antimicrobial effectivity testing are combined with an additional method to correctly differentiate between viable, dead, and VBNC cells.

#### 3.2.2. EMA/PMA-PCR

In the field of membrane-based detection methods, the polymerase chain reaction (PCR) combined with DNA intercalating dyes is a highly rapid and, in combination with real-time amplification, a quantitative (qPCR) method for detecting viable and VBNC cells. In contrast, for classic PCR or qPCR assays without DNA intercalating dyes, it is not possible to differentiate dead from viable bacterial cells causing free DNA in PCR reagents or DNA from dead cells leading to false-positive signals [90,91,92,93].

As bacterial cells in the VBNC state are scarcely detectable using culture-based methods, a variety of PCR and qPCR applications combined with DNA intercalating dyes have been established in recent years for a selective detection of viable cells of human pathogens from the food and environment, as listed in Table 1.

The addition of DNA intercalating dyes such as propidium monoazide (PMA) or ethidium monoazide (EMA) during the sample analysis procedure is an easy-to-perform step prior to PCR. The principle is based on the covalent bonding of dyes to freely accessible DNA and DNA from membrane-compromised dead cells via photoactivation. The obtained photo-inducible azide groups inhibit the DNA amplification [91,144]. EMA in contrast to PMA can also penetrate viable cells of some bacterial species due to a lower charge. Metabolically active cells export EMA via transport pumps actively or passively via diffusion barriers out of the cell. Nevertheless, the remaining chemical residues in viable cells lead to a substantial loss of DNA resulting in false-negative results or inaccurate quantification [100,144,145,146]. However, the organic azides from unbound dyes interfere with the DNA polymerase during amplification and lead to a decrease in the PCR reaction efficiency. For the use of EMA and PMA, around 25% of organic azides were reported to remain unbound in a PCR reaction [147]. Therefore, using an optimal adjusted dosage of the dyes in combination with longer templates (sequences longer than 600 base pairs) the ΔCq value comparing Cq values with and without DNA intercalating dyes increase. This is due to the fact that the DNA polymerase is less affected from unbound dyes and the dyes strongly inhibit exogenous DNA and DNA from membrane-compromised dead cells. Furthermore, longer target sequences allow the usage of lower dye concentrations. The disadvantage targeting long amplicons and DNA intercalating dyes is the effect of an increased Cq value caused by a longer amplicon time. The most important benefit targeting long amplicons is to suppress false-positive signals for a broad range of EMA/PMA-PCR assays [107,144,147,148].

Universal primers amplifying long target sequences within conserved genomic regions in combination with DNA intercalating dyes allow the simultaneous detection and antimicrobial effectivity testing of a wide spectrum of VBNC cells from different species. Schnetzinger et al. (2013) described a PMA-qPCR assay with a target sequence of 1108 bp within the 16S ribosomal RNA (rRNA) region for detecting the species relevant in bioburden determination in the pharmaceutical industry [147]. Soejima et al. (2011) published an EMA-qPCR assay of long DNA templates within the 16S rRNA (1490 bp) and 23S rRNA (2840 bp) regions, which can target a broad range of *Enterobacteriaceae* relevant for testing pasteurized milk [107].

S. Lee and Bae (2018) used the PMA-qPCR assay for viability testing of VBNC *Pseudomonas* (*P.*) *aeruginosa* cells induced by an antibiotic exposure of different types of antibiotics. They showed that aminoglycosides such as gentamicin, which cause protein synthesis inhibition by targeting the 30S rRNA region, induce the VBNC state [29]. Furthermore, the induced VBNC cells persist over 24 h under the antibiotic treatment without a reduction in the cell number. Therefore, the treated cells of *P. aeruginosa* PAO1 with 12 and 24 µg mL^−1^ gentamycin were identified as viable in the PMA-qPCR and unchanged in their ΔCt values over 24 h indicating that the cell membrane might be still intact. In addition, the intact cells were not detectable on culture-based methods over the time meaning that the cells were potentially identified as VBNC cells, which are resistant against gentamycin concentrations up to 24 µg mL^−1^ over 24 h [29]. Moreover, Kobayashi et al. (2010) reported for *S. aureus* and *S. epidermidis* constant positive ΔCt values after 24 h under the treatment with gentamycin indicating that the number of viable cells was still present [30].

PCR assays in combination with DNA intercalating dyes are an “end-point” method for detecting target gene sequences of viable bacterial cells and are currently used in combination with gel electrophoresis, quantitative real-time PCR (qPCR), reverse transcription PCR (rt-PCR), isothermal amplification PCR assays up to next-generation sequencing applications [149,150,151]. In contrast to stable DNA based applications, RNA degrades rapidly after the cell death, which makes it more challenging to handle. Messenger RNA is only produced by metabolically active cells indicating viable cells. Lu et al. (2015) observed in VBNC cells of *V. parahaemolyticus* RIMD2210633 an upregulation of genes related to non-metabolic functions and a downregulation of genes involved in the metabolic activity compared to exponential and early stationary phase cells [152]. Nevertheless, mRNA degradation can occur by RNA-degrading enzymes during sampling and storage resulting in false-negative signals [153]. On the other hand, mRNA is stable for an unknown period of time so that false-positive results may occur [154].

Applicability for antimicrobial effectivity testing:

PCR in combination with DNA intercalating dyes is a highly rapid, species specific, and sensitive method for the quantification of viable and VBNC cells with an intact cell membrane, while free DNA and cells with a compromised cell membrane are not detected. PCR applications can be applied after an evaluating step using control strains in already existing processes. Existing protocols are applied to a wide variety of bacterial species detecting a wide range of genetic target sequences. In addition to specific bacteria, gene targets within conserved genomic regions allow a simultaneous detection of a broad range of bacterial species from food and environmental samples. The addition of DNA intercalating dyes to samples as a pre-treatment step before the DNA extraction is simple to perform and quantitative results using real-time amplification can be obtained via standard curves including the target gene sequence. Results can be specified in log copy numbers, as well as a log concentration. Furthermore, the efficiency of bactericides on culturable bacterial cells and cells in the VBNC state can be quantified by ΔCq values calculated through subtracting the Cq value before and after the treatment. When combined with the plate count method for quantification of culturable cells, the methodological approach can be used to quantify viable, dead, and VBNC cells and for antimicrobial effectivity testing.

Limitations:

As for fluorescence microscopy, EMA/PMA-PCR assays measure the integrity of the cell membrane and could have limitations for discriminating VBNC cells obtained from conditions that damage the cell membrane. On the one hand, an intact cell membrane is not always an unequivocal identification for viability and provides no information on the metabolic activity of the cells. Therefore, some treated bacterial cells might seem to remain viable using EMA/PMA-PCR assays having an intact membrane, thus being impermeable to EMA or PMA, but are already dead at the metabolic level with the loss of membrane integrity occurring only after hours or days. A rapid loss of viability by inducing DNA damage followed by a slow decay of cellular components without directly affecting the cytoplasmic membrane can lead to false-positive signals. In this case, membrane damage is a secondary effect with an unknown time span when cells are susceptible to the dye uptake [33,91,93]. This effect is described for physical inactivation methods including UV-C decontamination [94,126,155,156,157], solar light-initiated photoinactivation [158,159,160], and low-temperature pasteurization [111,161,162,163], as well as for the antibiotic treatment with aminoglycosides, macrolides, rifampycin, and quinolones [30,93].

On the other hand, sublethal damaged bacterial cells with a perforated membrane lead to a false-negative result causing an inhibited DNA amplification considered by the uptake of the DNA intercalating dyes [67,154]. Lee and Bae (2018) observed that antibiotics, which interfere with the cell wall and periplasm membrane synthesis such as carbenicillin and colistin, might affect the membrane impermanent dyes that intercalate to DNA from membrane-compromised cells considering dead cells [29]. Kobayashi et al. (2010) showed similar results using PMA-qPCR to identify viable *S. aureus* and *S. epidermidis* after a treatment with vancomycin inhibiting a substantial proportion of PCR amplification [30]. For these reasons, that DNA intercalating dyes differentiate between membrane intact and membrane compromised cells, the method might be incapable for detecting VBNC cells from other membrane damaging conditions such as cationic surfactants [163,164], oxidative disinfectants, e.g., chlorine and ozone [117,163], as well as organic solvents and heat [162,163].

An additional limitation of the viability PCR is the time investment to establish and evaluate the method as a diagnostic tool. The necessary optimizing steps include sample preparation, internal amplification controls, as well as standard curves to estimate the detection limit. The mentioned parameters have to be established and evaluated for every individual gene target or when chemicals in the PCR reaction are replaced [78].

### 3.3. Active Metabolism-Cellular and Metabolic Properties as a Biomarker for Viable Bacteria and Resistance

Without the presence of cellular metabolism or energy, a cell (with the exception of spores) can be considered dead and therefore, it comes as no surprise, that these parameters have been widely used for the viability assessment of bacterial cells in the VBNC state. There have been efforts of using the uptake and subsequent degradation of fluorescent 2-[N-(7-nitrobenz-2-oxa-1,3-diazol-4-yl)amino]-2-deoxy-D-glucose (2-NBDG), which is comparable to glucose, to measure the active cell metabolism [70,165]. However, the vast majority of studies involving VBNC cells have used either the respiratory activity, or the presence/production of ATP as a measure to determine cell viability and will be the focus in the subsequent chapters.

#### 3.3.1. Cellular Energy

Adenosine triphosphate (ATP) is the energy source for metabolic activities in all living organisms and can be used as a viability indicator [23,24]. Many commercially available ATP detection kits have been used for cell viability determination for decades, including in the food industry, for drug discovery, assessment of drinking water quality, or in soil samples [50,126,166,167,168].

The application typically involves the addition of a reagent to lyse cells and release ATP, which, in the presence of luciferase, reacts with the substrate, D-luciferin, to produce light. The light intensity is then measured as relative light units (RLU), which is interpreted as a measure of ATP concentration [50,169]. It is difficult to establish a correlation between the total number of cells and RLU to provide a reasonable estimate of cellular activity. However, if combined with other tools such as live/dead staining, the concentrations of bacterial ATP in different aquatic microbial communities, for example, correlate well with the concentrations of living cells [168].

**Figure 6:**
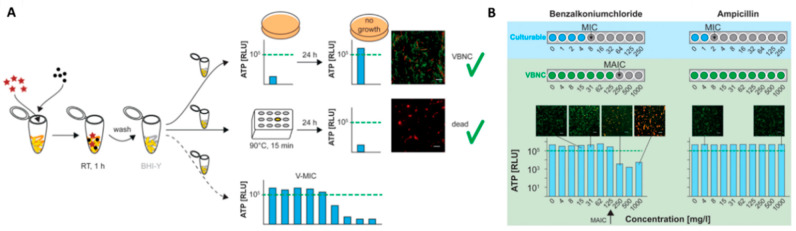
Workflow of the VBNC-minimum inhibitory concentration (MIC) assay, adapted from [170]. (**A**) The VBNC state is induced by a specific combination of detergent (stars) and salt (dots); after induction, cells are washed and resuspended in a fresh Brain Heart Infusion (BHI) medium; positive control: Untreated VBNC cells confirmed by ATP measurements and BacLight™ viability assay; negative control: VBNC cells killed at 90 °C for 15 min; minimum ATP inhibitory concentration (MAIC) of antimicrobials is determined by measuring the *de novo* ATP production of VBNC cells exposed to the respective antimicrobial, viability marker: 10^5^ relative light units (RLU) threshold. (**B**) VBNC-MIC assay MIC determination for culturable and MAIC determination for VBNC *L. monocytogenes* against benzalkonium chloride and ampicillin.

For this reason, in the VBNC research ATP is a popular biomarker for microbial viability as it is a fast, cheap, and easy approach to distinguish the viable from dead cells [12,27,170]. In 2019, Robben et al. [170] compared the *de novo* ATP production of five different pathogens (*L. monocytogenes*, *E. coli*, enteropathogenic *E. coli*, *Salmonella enterica* subsp *enterica* serovar Typhimurium, *and S. aureus)* in the VBNC state, to plate count during exposure to different heat stress and demonstrated that the ATP production of VBNC cells was in agreement with the plate count of culturable bacteria. On a laboratory scale, this approach can be used to determine the minimum ATP inhibitory concentration, which is a VBNC-MIC testing approach (Figure 6). After induction of the VBNC, state cells are exposed to a dilution series of antimicrobial compounds and incubated with species-specific conditions. To avoid overestimation of the total ATP counts, extracellular ATP has to be removed by filtration of the cell suspension, enzymatic hydrolysis of extracellular ATP, or washing steps prior to an overnight incubation of the VBNC cells. Subsequently, the *de novo* ATP production can be determined for antimicrobial effectivity testing and a cut-off in ATP production shows the minimum ATP inhibitory concentration (MAIC) for VBNC cells. Using this approach, the authors could demonstrate that bacteria in the VBNC state showed elevated tolerance levels against antibiotics, as well as disinfectants, while no differences were found in regard to heat stress. As the authors only compared MIC and MAIC results, but did not determine the minimal bactericidal concentration (MBC), it cannot be completely excluded that MBC and MAIC coincide.

Applicability for antimicrobial effectivity testing:

The methodological approach based on the *de novo* ATP production is able to qualitatively detect metabolically active cells including viable and VBNC cells. VBNC cells are defined by a significant *de novo* ATP production without the occurrence of culturable cells. The approach is not able to detect viable and VBNC cells at the same time from the same sample and dead cells are not actively detected, but rather inferred by the reduced *de novo* ATP production. The utilization of the *de novo* ATP production as a viability indicator offers the chance to perform a high throughput end-point screening for antibiotic and antimicrobial effectivity testing based on the *de novo* ATP production high-throughput in a similar setup as the established micro-broth dilution assay. Especially the fact that the *de novo* ATP production is a universal viability parameter for all bacterial species, makes the methodological approach very promising, as it should be readily transferable to other target organisms without additional adaptations. The approach is not dependent on the successful resuscitation of the target organisms, but as is the case for cell proliferation assays (see Section 3.1), is susceptible to the possible regrowth of culturable cells.

Limitations:

Non-microbial sources of ATP interfere with ATP measurements and may lead to false-positive results, which limit the application to laboratory research and is hardly applicable in industrial environments. Using solely ATP measurements, it is difficult to distinguish between resuscitation and regrowth. Therefore, the use of adequate controls is essential to make sure the VBNC induction is complete and includes the whole population. As there are also possible signals from dying cells or other interferences, a quantitative analysis is difficult without additional cell counting methods.

#### 3.3.2. Respiratory Activity and Membrane Potential

The respiratory activity depends on a functioning electron transport chain, which is the main process for maintaining a membrane potential. Therefore, the presence of the respiratory activity is a viability indicator, linking the membrane potential and the recycling of reducing equivalents which are produced in many catabolic reactions [83,168].

There are different fluorescence staining methods available to measure the metabolic activity via an active efflux pump system, enzyme activity, or an intact membrane potential [83]. Emerson et al. [23] described a double staining assay with the anionic fluorescent molecule Alexa Fluor™ hydrazide (AFH) and the cationic fluorescent molecule diethyloxacarbocyanine iodide DiOC2 (3) or SYBR Green I to measure the membrane potential. AFH binds on carbonyl groups of damaged proteins accessible from membrane compromised aging, dying, and dead cells resulting in a blue fluorescence signal. The counter staining with DiOC2 (3) or SYBR Green I shows an intact membrane potential when the molecule enters the cell from a positive gradient outside to a negative gradient inside the cell resulting in a green fluorescence signal [23,171]. Li et al. [16] described a single staining method with the *p*-iodonitrotetrazolium violet (INT) to measure the uptake of the molecule through the cell membrane via an active electron transport system. Metabolically active cells reduce tetrazolium to a red-fluorescent formazan that accumulates within the cells and is detectable by microscopy or cytometry. The same principle can be measured using CTC, which is reduced to red-fluorescent 3-cyan-1,5-ditolyl formazan (CTF) [25,73,172,173]. Using the counterstaining with DAPI, the total bacterial count emit in a red and blue fluorescence signal, whereas the viable metabolic active cells show a red fluorescence signal [65,66]. An example to measure the enzyme activity is described by Yoshioka et al. [165] using the dye 2-NBDG. The fluorescent 2-NBDG molecule uptake into the cell is comparable to glucose and the green fluorescence signal degraded continuously by an active cell metabolism [70,165].

The different fluorescence staining methods can easily be detected by fluorescence microscopy and/or flow cytometry [174]. In addition, the use of flow cytometry enables rapid, in situ analysis of single cells, including viruses [175], and in combination with additional fluorescent staining techniques such as membrane integrity staining (see Section 3.3.1), quantitative as well as qualitative data can be obtained with continuous measurements. A high number of cells can be sorted and enumerated within a few seconds. Until now, flow cytometry has been used mainly in the field of clinical research [176,177]. The detection and definition of bacterial viability have been the major microbiological issues in cytometry [178]. Using flow cytometry, it has recently been shown that four different physiological states of bacteria can be distinguished: Reproductively viable, metabolically active, intact, and permeabilized [179,180], but rapid approaches to distinguish between VBNC and dead cells and especially their use of antimicrobial effectivity testing are rare until now.

**Figure 7:**
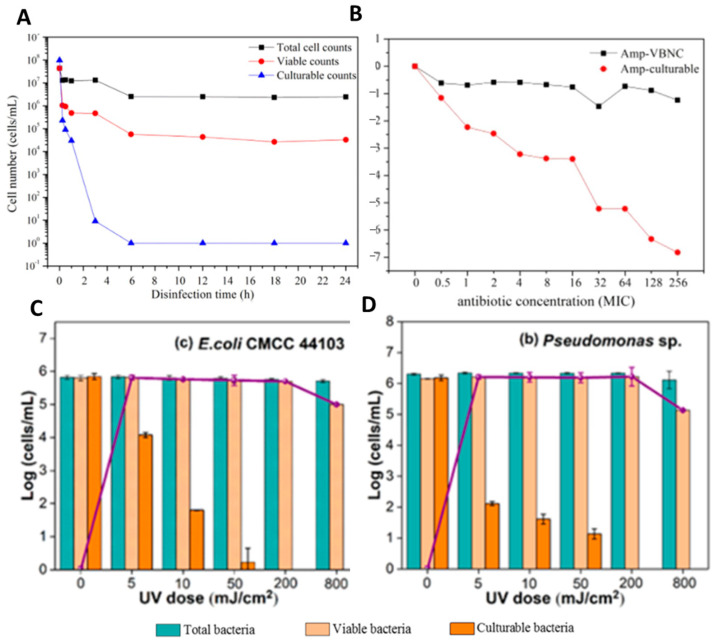
CTC flow cytometry used for tolerance testing of *E.coli*, adapted from [47,181]. (**A**) Entry of *E. coli* into the VBNC state on incubation by chlorination at the dosage of 0.5 mg/L free chlorine. Shown are total cell counts (black square), culturable counts (blue triangle), and viable counts (red circle). (**B**) Persistence of *E. coli* exposed to ampicillin. Black circles represent *E. coli* in the VBNC state, while red circles represent *E. coli* in the culturable state. (**C**,**D**) Total, viable, and culturable cell counts of *E. coli* (**C**) and *Pseudomonas* spp. (**D**) after the treatment by various UV fluences. The purple line represents the number of VBNC bacteria, determined as the difference between the number of viable and culturable cells.

Lin et al. [47] and Guo et al. [181] showed that the CTC flow cytometry is a suitable method to perform antibiotic effectivity testing and also for evaluating the UV disinfection and chlorine-based disinfection (Figure 7).

They found that CTC flow cytometry characterizes the essential viability of VBNC cells although CTC is only involved in a small part of the respiratory chain and reflects only the redox ability of the electron transport chain. Overall, the authors could demonstrate the effectiveness of their methodological approach to perform antimicrobial effectivity testing against bacteria in the VBNC state [47,181].

Applicability for antimicrobial effectivity testing:

The methodological approach based on detecting the respiratory activity and membrane potential can be used to quantify viable as well as VBNC cells. By combining the approach with plate count methods, the amount of culturable as well as VBNC cells can be quantified from the same sample. In combination with additional staining methods (e.g., whole cell or membrane integrity), the approach can be used to accurately distinguish and quantify viable, dead, and VBNC cells. The combination of flow cytometry and fluorescent dyes for the detection of the respiratory activity and membrane potential offers the potential to perform antimicrobial effectivity testing of VBNC cells. In contrast to ATP detection methods, this approach can obtain quantitative results and is not susceptible to the possible regrowth of culturable cells.

Limitations:

A considerable disagreement exists to the exact value of this method for the analysis of environmental samples [168]. One of the shortcomings of the CTC assay is that it often requires long staining times (up to 24 h), which makes it less interesting for a rapid microbiological assay. Such a long staining time could result in other viability changes (e.g., die-off, regrowth) in the samples [144,182]. In addition, for flow cytometry, it is essential that the crystals form inside the cells, which is not always the case. Moreover, it has been noted that not all bacteria are capable of reducing formazan salts (since not all bacteria have a functioning electron transport chain), which raises questions about the general applicability of the method outside laboratory environments.

## 4. Summary and Conclusions

In recent years, the awareness about the viable but non-culturable state of bacterial pathogens has dramatically increased and the fact that bacteria in the VBNC state are not visible for routine, growth based detection methods led to the development of novel analytical methods. The majority of methodological approaches determines at least one of the generally accepted viability parameters such as resuscitation, membrane integrity, metabolic activity, or a combination thereof. Thanks to these new methodological approaches, it was discovered that, due to their low metabolic activity and cellular changes in the VBNC state, bacteria have a drastically increased tolerance against antibiotics, as well as antimicrobials and that the methodological approaches could be utilized for antimicrobial effectivity testing against VBNCs.

The majority of methodological approaches were originally developed with a clear focus on the rapid and cost-effective detection of VBNC pathogens and high-throughput methods for antimicrobial effectivity testing are still rare. However, it is our opinion that the relevance of the topic will facilitate their development. As reviewed in this article, there have been several different methodological approaches in order to investigate the tolerance and resistance of VBNC cells, measuring one viability parameter (cell proliferation, cell membrane integrity, or metabolic activity). Based on their underlying experimental principles and the currently available literature, each of the methodological approaches has certain advantages, as well as limitations and for antimicrobial effectivity testing the respective method has to be chosen carefully (Table 2).

What also becomes clear from the current literature is a lack of comparative studies regarding the respective methods and their respective outcomes. To the best of our knowledge, there are no studies yet available which perform tolerance testing of bacteria in the VBNC state with different experimental approaches. Given the relative novelty of many of the developed methods this is not surprising, but we would highly encourage researchers to increase the collaboration on this topic. The aim should be to establish standard protocols or guidelines regarding VBNC tolerance testing and how they could ultimately complement existing and well-established procedures for culturable bacteria. Furthermore, standard protocols are needed to assess the impact of decontamination effectiveness against VBNC cells when novel decontaminating technologies are available. The application and development of new methods to further investigate the VBNC state and its tolerance is very important to generate new efficient intervention methods to ensure water and food safety to reduce public health risks.

## Figures and Tables

**Table 1:** PCR and qPCR propidium monoazide/ethidium monoazide (EMA/PMA) applications.

Bacteria	Application	Reference
***Aeromonas hydrophila***	PMA-pPCR	[94]
***Arcobacter butzleri*** ***Arcobacter* spp.**	PMA-qPCR PMA-qPCR	[95] [96]
***Bacillus cereus***	PMA-qPCR PMA-PCR	[97] [98]
***Brucella* spp.**	PMA-qPCR	[99]
***Campylobacter jejuni***	EMA-qPCR	[95,100,101,102]
***Campylobacter* spp.**	PMA-qPCR	[103,104,105]
***Clostridium perfringens***	PMA-qPCR	[97]
**Gram-negative bacteria**	PMA-qPCR	[106]
***Enterobacteriaceae***	PMA-qPCR	[97,107,108]
***Eenterococcus faecalis***	PMA-pPCR	[94]
***Enterococcus* spp.**	PMA-qPCR	[101]
***Escherichia coli***	EMA-qPCR PMA-PCR	[94,98,109,110,111,112,113] [114,115,116]
***Helicobacter pylori***	PMA-qPCR	[117]
***Legionella pneumophila***	PMA-qPCR	[118,119,120,121]
***Legionella* spp.**	EMA-qPCR PMA-qPCR	[122] [123,124]
***Listeria monocytogenes***	EMA-qPCR EMA/PMA-qPCR PMA-pPCR PMA-PCR	[109] [125] [100,126,127,128] [115]
***Pseudomonas aeruginosa***	PMA-pPCR	[29,126,129]
***Salmonella enterica* subsp. *enterica* serovar Enterica** ***Salmonella enterica* subsp. *enterica* serovar Typhimurium** ***Salmonellaenterica* subsp. *enterica* serovar Enteritidis** ***Salmonella* spp.**	PMA-PCR PMA-qPCRPMA-PCR PMA-qPCRPMA-qPCR EMA-qPCRPMA-PCR PMA-qPCR	[98] [95,115] [101,119,130] [98,109,131] [132,133,134,135,136]
***Staphylococcus aureus***	PMA-qPCR	[97,119,126,137]
***Vibrio cholerae***	PMA-qPCR	[138]
***Vibrio parahaemolyticus***	PMA-qPCR	[126,139,140,141]
***Vibrio vulnificus***	EMA-qPCR	[142,143]
***Yersinia enterocolitica***	PMA-qPCR	[95]

**Table 2:** Summary of the currently available methods for resistance screening for VBNCs.

Method	Viability Parameter	Target and Unit	Advantage	Limitation
**Resuscitation**	Cell proliferation	Entire cell [CFU]	Directly comparable with traditional methods No bias due to comparing different methods	Only very few reliable quantitative resuscitation models available Only with pure cultures in a laboratory setting possible
**Viability PCR/qPCR**	Membrane integrity	DNA [Gene copies]	Qualitative and quantitative results can be obtained Species data can be obtained from mixed (or environmental) samples	Susceptible to non-lethal membrane damages Time- and cost- intensive validation End-point method
			Capability to differentiate viable and dead cells	
**Fluorescence microscopy**	Membrane integrity	Membrane components and DNA [Cell numbers]	Stains can be used against a wide range of bacterial species Potential for high-throughput analysis in combination with flow cytometry Can be combined with other florescence dyes (e.g., for active metabolism) Capability to differentiate viable and dead cells	No differentiation between bacterial species Susceptible to non-lethal membrane damages
**Cellular** **energy**	Active metabolism	ATP [RLU]	Species independent analysis Potential for high-throughput analysis	No differentiation between bacterial species Only with pure cultures in a laboratory setting possible
**Respiratory activity**	Active metabolism	Reduction of fluorescencent dyes [Cell numbers]	Stains can be used against a wide range of bacterial species High-throughput analysis in combination with flow cytometry Automated analysis Can be combined with other florescence dyes (e.g., for membrane integrity)	No differentiation between bacterial species Not all bacterial species can be analyzed
